# Cooperation-Controlled Learning for Explicit Class Structure in Self-Organizing Maps

**DOI:** 10.1155/2014/397927

**Published:** 2014-09-18

**Authors:** Ryotaro Kamimura

**Affiliations:** IT Education Center and School of Science and Technology, 1117 Kitakaname, Hiratsuka, Kanagawa 259-1292, Japan

## Abstract

We attempt to demonstrate the effectiveness of multiple points of view toward neural networks. By restricting ourselves to two points of view of a neuron, we propose a new type of information-theoretic method called “cooperation-controlled learning.” In this method, individual and collective neurons are distinguished from one another, and we suppose that the characteristics of individual and collective neurons are different. To implement individual and collective neurons, we prepare two networks, namely, cooperative and uncooperative networks. The roles of these networks and the roles of individual and collective neurons are controlled by the cooperation parameter. As the parameter is increased, the role of cooperative networks becomes more important in learning, and the characteristics of collective neurons become more dominant. On the other hand, when the parameter is small, individual neurons play a more important role. We applied the method to the automobile and housing data from the machine learning database and examined whether explicit class boundaries could be obtained. Experimental results showed that cooperation-controlled learning, in particular taking into account information on input units, could be used to produce clearer class structure than conventional self-organizing maps.

## 1. Introduction

### 1.1. Visualization by Self-Organizing Maps

The present method aims to improve visualization performance and in particular to produce clear class structure for the self-organizing maps. Thus, we will begin with a brief introduction to the visualization problems found in self-organizing maps. The self-organizing map, or SOM [1, 2], is one of the most important techniques in the study of neural networks. It has been widely used as one of the basic techniques of visualization. However, one of the problems in SOMs concerns the representation of SOM knowledge for visual inspection and interpretation. Though the SOM has good reputation in visualization, it is actually difficult to visualize and interpret its knowledge.

To facilitate visualization, usually, final results obtained by conventional SOMs are accompanied by certain visualization methods. For example, to extract the characteristics of prototype vectors, the U-matrix and its variants [3, 4] are commonly used, which compute distances between each map unit and neighboring ones. The U-matrix is a convenient tool for showing class boundaries on the output map, though it is not good at extracting finer class boundaries [5]. Moreover, the properties of prototype vectors can be examined by plotting component planes along all input variables. The inspection of component planes shows the spread of values of the components as well as correlations between the corresponding input patterns [6]. In the detection of the overall cluster structure, usually linear and nonlinear dimensionality reduction methods such as the principal component analysis (PCA) [7], Sammon map [8], and many other nonlinear methods [9–11] can be used to represent the prototype vectors in a lower dimension. In addition, the responses to data samples can be examined. The responses are usually based upon the best matching unit (BMU) of the data sample. The data histogram is also used to convey the knowledge in SOM for multiple vectors [6]. The surface response shows the relative worth of each unit in representing the data sample [6]. Recently, advanced visualization techniques such as gradient field and borderline visualization techniques based on the vector field [12] have been developed. These can be used to clarify a class structure or class boundaries using the vector fields, showing homogenous areas. The connectivity matrix of prototype vectors between the BMU (best-matching unit) and the second BMU is used to guide the accurate capture of cluster boundaries [5]. The gradient-based SOM matrix was introduced to clarify cluster boundaries by strengthening distances with low activity [13].

While the above techniques are applied to SOM results, there are several methods where connection weights are actively modified so as to improve class structure. For example, the visualization induced self-organizing map (ViSOM) [14] was introduced to preserve data structure and topology as faithfully as possible by regularizing the distances of interneurons. Along the same line, the probabilistic regularized self-organizing map (PRSOM) [15] was also proposed to regularize interneuron distances by introducing the MDS type metric to preserve pair-wise distances between neurons in the input and output space. The double self-organizing feature map (DSOM) [16] is a heuristic method of adjusting neurons' position vectors such that neurons with similar responses to input patterns are close to each other. The polar self-organizing map (PolSOM) [17] and its variant, the probabilistic polar self-organizing map (PPoSOM) [18], aim to visualize data on a two-dimensional polar coordinate map by measuring the radius and angle instead of the conventional Cartesian coordinates. PPoSOM is claimed to be particularly good at visualizing differences between two neurons on the new coordinates.

### 1.2. Cooperation-Controlled Learning

In the above section, we listed a number of visualization techniques which can be used to simplify the interpretations of final representations obtained by the conventional SOM, and, in particular, to clarify the cluster structure in SOMs. These are only a few examples of the many visualization techniques developed for conventional SOMs. Though there are a number of visualization techniques, we have still had serious problems in interpreting final representations in SOM because of the complex and ambiguous representations therein. In particular, class or cluster boundaries cannot be easily extracted.

The generation of these ambiguous class boundaries in the conventional SOM is, from our point of view, due to the fact that the main focus of the conventional SOM is on cooperation among neurons. The method has been developed so as to make cooperation between neurons more effective. For example, the good performance of self-organizing maps has been overwhelmingly evaluated by the trustworthiness and continuity in the output and input spaces [19–25]. No attempts have been made to evaluate the good performance of self-organizing maps on the clarity of the obtained class structure. In addition, we can infer that attention to cooperation seems to be accompanied by the side effect of neighboring neurons behaving in the same way, even if class boundaries among neurons exist. This means that the class boundaries become weaker as the effect of cooperation becomes dominant, and neighboring neurons become similar to each other. We might infer from this that cooperation among neurons and the detection of class boundaries are contradictory to each other. As we take into account cooperation between neurons and continuity in the output space in SOMs, naturally class boundaries or discontinuity should be reduced as much as possible.

Thus, we need to control or reduce the effect of cooperation among neurons; much more attention should be paid to the extraction of explicit class boundaries. To control cooperation, we here introduce multiple points of view in neural networks. Components seen from multiple points of view can interact with each other to have special effects on learning in neural networks. We here restrict ourselves to a case where a network is viewed from two points of view. Namely, we consider a network viewed from individual and collective points of view, for the sake of easy implementation and explanation. One form of neural network is called a “cooperative network,” in which neurons are treated collectively. The other network is called an “uncooperative network,” where neurons are treated individually. The interaction between the two types of networks is controlled by the cooperation parameter. The learning method to control the interaction of cooperative and uncooperative networks is called “cooperation-controlled learning.” Cooperation-control learning can be used to improve the visualization performance of self-organizing maps. In self-organizing maps, cooperation between neurons tends to cover class boundaries. These can be uncovered by enhancing the differences between neurons by making a distinction between the collective and individual behaviors of the neurons.

### 1.3. Outline

In Section 2, we introduce “cooperation-controlled learning,” where cooperative and uncooperative networks interact with each other. We first explain the concept of multiple points of view by focusing upon two types of networks. Then, we show the general form of cooperative-controlled learning with three types of learning, namely, uncooperative, unweighted, and weighted cooperation-controlled learning. The computational methods of all the network types are explained, where mutual information between input patterns and competitive units is maximized in terms of free energy minimization. The actual computational procedure is composed of two steps. In the first step, simplified cooperative learning is applied, where only cooperative networks are taken into account to simplify learning and to borrow the conventional learning procedures from SOMs. In the second step, weighted and unweighted cooperation-controlled learning is applied. In Section 3, we consider the automobile and housing data from the machine learning database. Our main objective is to experimentally demonstrate the differences between the different types of cooperative learning and cooperation-controlled learning. In particular, we stress that, by using the weighted cooperation-controlled learning, including information on input units, clear class boundaries can be detected. The explicit class boundaries are in particular generated when the role of uncooperative networks is large.

## 2. Theory and Computational Methods

In this section, we first explain our cooperation-controlled learning in a general sense. We then present three computational methods, namely, uncooperative learning and two types of cooperation-controlled learning (unweighted and weighted cooperation-controlled learning). Finally, the actual computational procedures for cooperation-controlled learning are described.

### 2.1. Cooperative and Uncooperative Network

**Figure 1:**
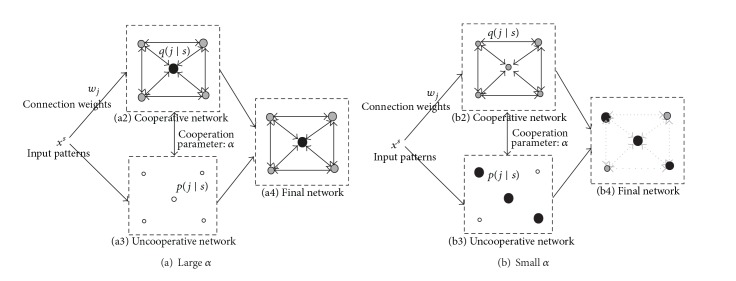
Concept of cooperation-controlled learning.

Cooperation-controlled learning is based upon the supposition that a neural network should be examined from different points of view. In this study, these points of view are cooperative and uncooperative networks. In the uncooperative network, each neuron is treated individually, while in the cooperative network each neuron is treated collectively. A final network is obtained by the interaction of these two types of networks. Two examples of interaction are shown in Figure 1. The importance of cooperative and uncooperative networks is supposed to be determined by the cooperation parameter *α*. When the parameter *α* is large in Figure 1(a), the cooperative network is more influential. As shown in Figure 1(a2), the centrally located neuron fires very strongly, while in Figure 1(a3), which shows the uncooperative network, all the neurons fire weakly. A final network by interaction reflects the state of the cooperative network in Figure 1(a4). When the cooperation parameter *α* is small, an uncooperative network is more important. In Figure 1(b3), three neurons fire very strongly in the uncooperative network. On the other hand, all the neurons fire very weakly in Figure 1(b2). Thus, the state of the uncooperative network is reflected in the final network in Figure 1(b4).

We introduce this interaction because we try to produce explicit class structure in self-organizing maps. The self-organizing map is a well-known technique for the purpose of visualization. For example, to visualize class structure, we must produce explicit class boundaries. However, it is difficult to visualize class boundaries in self-organizing maps. As discussed in Section 1, there have been many attempts to visualize connection weights [3, 6, 8, 14, 16, 17, 26–28]. One of the main problems lies in the focus upon cooperation between neurons in the conventional self-organizing maps; neighboring neurons must behave as similarly as possible. This behavior is thought to contribute to the difficulty in visualizing class boundaries. Even in the class boundaries, neurons are forced to change gradually and to reduce discontinuities as much as possible. Cooperation-controlled learning aims to control the degree of cooperation. When it is applied to the self-organizing maps, the cooperation can be weakened, which makes class boundaries more explicit.

### 2.2. General Cooperation-Controlled Learning

Let us explain basic procedures for cooperation-controlled learning. Now, the *s*th input pattern of total *S* patterns can be represented by

(1) $$\begin{array}{c} x^{s}={\left[x_{1}^{s},x_{2}^{s},\ldots ,x_{L}^{s}\right]}^{T},\;s=1,2,\ldots ,S. \end{array}$$

Connection weights into the *j*th competitive unit of total *M* units are computed by

(2) $$\begin{array}{c} w_{j}={\left[w_{j1},w_{j2},\ldots ,w_{jL}\right]}^{T},\;j=1,2,\ldots ,M. \end{array}$$

As shown in Figure 1, supposing that the probability *p*(*j*∣*s*) for competitive units in an uncooperative network and *q*(*j*∣*s*) denote the firing probability for a cooperative network, then we must decrease the following KL divergence measure:

(3) $$\begin{array}{c} D=\overset{S}{\underset{s=1}{\sum }}\;\overset{M}{\underset{j=1}{\sum }}p\left(s\right)p\left(j\;|\;s\right)\log\frac{p\left(j\;|\;s\right)}{q\left(j\;|\;s\right)}. \end{array}$$

In minimizing the KL divergence, we suppose that the corresponding cost *E* of assigning input patterns to competitive units is represented by quantization errors between input patterns and connection weights:

(4) $$\begin{array}{c} E=\overset{S}{\underset{s=1}{\sum }}\;\overset{M}{\underset{j=1}{\sum }}p\left(s\right)p\left(j\;|\;s\right){||x^{s}-w_{j}||}^{2}. \end{array}$$

When this quantization error is fixed, the optimal firing probability p*(*j*∣*s*) to minimize the KL divergence is obtained by

(5) p∗(j ∣ s) =q(j ∣ s)exp⁡{−(1/2)(xs−wj)TΛ(xs−wj)}∑m=1Mq(j ∣ s)exp⁡{−(1/2)(xs−wm)TΛ(xs−wm)},

where the *L* × *L* matrix Λ is called a “scaling matrix,” and the *kl*th element of the matrix denoted by (Λ)_*kl*_ is defined by

(6) $$\begin{array}{c} {\left(\Lambda \right)}_{kl}=\delta_{kl}\frac{p\left(k\right)}{\sigma^{2}},\;k,l=1,2,\ldots ,L, \end{array}$$

where *σ* is the spread parameter and *p*(*k*) shows the firing probability of the *k*th input unit.

By putting the optimal firing probability p*(*j*∣*s*) into the KL divergence, we obtain the free energy:

(7) F=∑s=1Sp(s)∑j=1Mp∗(j ∣ s)||xs−wj||2 +2σ2∑s=1Sp(s)∑j=1Mp∗(j ∣ s)log⁡p∗(j ∣ s)q(j ∣ s)=−2σ2∑s=1Sp(s) ×log⁡∑j=1Mq(j ∣ s)exp⁡{−12(xs−wj)TΛ(xs−wj)}.

By differentiating the free energy, we can obtain the reestimation formula:

(8) $$\begin{array}{c} w_{j}=\frac{\sum_{s=1}^{S}p\left(j\;|\;s\right)x^{s}}{\sum_{s=1}^{S}p\left(j\;|\;s\right)}. \end{array}$$

### 2.3. Typology of Cooperation-Controlled Learning

To summarize, we have two types of learning, namely, uncooperative and cooperation-controlled learning. All methods can be obtained by putting different *q*(*j*∣*s*) values into the KL-divergence. First, the most fundamental learning is uncooperative learning, where units are individually treated and do not cooperate with each other. In cooperation-controlled learning, we can distinguish between two types of cooperation-controlled learning, namely, unweighted and weighted cooperation-controlled learning, by considering whether we take into account the information on input units. In unweighted cooperation-controlled learning, relations between uncooperative and cooperative networks are considered, but information on input units is not taken into account. In weighted cooperation-controlled learning, in addition to cooperative and uncooperative networks, the firing probabilities of input units (variables) are taken into account. The main objective of this paper is to experimentally demonstrate the effectiveness of the cooperation-controlled learning and, in particular, the difference between the two types of learning, namely, unweighted and weighted cooperation-controlled learning.

### 2.4. Uncooperative Learning

Uncooperative learning is a method in which neurons do not cooperate with each other, as shown in Figure 1(a3). In actual implementation, the method corresponds to our information-theoretic competitive learning [29–31]. In this method, competition processes are supposed to be realized by maximizing mutual information between competitive units and input patterns. In this learning, we suppose that *q*(*j*∣*s*) is equi-probable, and the KL-divergence becomes

(9) D=∑s=1S ∑j=1Mp(s)p(j ∣ s)log⁡p(j ∣ s)q(j ∣ s)=log⁡M+∑s=1S ∑j=1Mp(s)p(j ∣ s)log⁡p(j ∣ s).

Thus, KL-divergence minimization corresponds to entropy maximization. By minimizing mutual information or maximization conditional entropy, we have

(10) $$\begin{array}{c} p^{*}\left(j\;|\;s\right)=\frac{\exp\left\{-\left(1/2\right){\left(x^{s}-w_{j}\right)}^{T}\Lambda \left(x^{s}-w_{j}\right)\right\}}{\sum_{m=1}^{M}\exp\left\{-\left(1/2\right){\left(x^{s}-w_{m}\right)}^{T}\Lambda \left(x^{s}-w_{m}\right)\right\}}. \end{array}$$

By inputting these optimal probabilities, we have the free energy:

(11) $$\begin{array}{c} F=-2\sigma^{2}\overset{S}{\underset{s=1}{\sum }}p\left(s\right)\log\overset{M}{\underset{j=1}{\sum }}\exp\left\{-\frac{1}{2}{\left(x^{s}-w_{j}\right)}^{T}\Lambda \left(x^{s}-w_{j}\right)\right\}. \end{array}$$

By differentiating the free energy, we obtain

(12) $$\begin{array}{c} w_{j}=\frac{\sum_{s=1}^{S}p^{*}\left(j\;|\;s\right)x^{s}}{\sum_{s=1}^{S}p^{*}\left(j\;|\;s\right)}. \end{array}$$

### 2.5. Unweighted Cooperation-Controlled Learning

An uncooperative network, or a network without cooperation between units, tries to imitate the cooperative network or network with cooperation. For the cooperative network, in the training mode, we first try to borrow the computational methods developed for the conventional self-organizing maps, and then use the ordinary neighborhood kernel used for SOM, namely,

(13) $$\begin{array}{c} h_{jc}\propto \exp\left(-\frac{{||r_{j}-r_{c}||}^{2}}{2\sigma_{\text{nh}}^{2}}\right), \end{array}$$

where **r**_*j*_ and **r**_*c*_ denote the position of the *j*th and *c*th units in the output space. The cooperative outputs can be defined by the summation of all neighboring competitive units:

(14) q(j ∣ s) =∑c=1Mhjcexp⁡{−(1/2)(xs−wc)TΛ(xs−wc)}∑m=1M∑c=1Mhmcexp⁡{−(1/2)(xs−wc)TΛ(xs−wc)},

where the *kl*th element of the scaling matrix (Λ_cp_)_*kl*_ is given by

(15) $$\begin{array}{c} {\left(\Lambda_{\text{cp}}\right)}_{kl}=\delta_{kl}\frac{p\left(k\right)}{\sigma_{\text{cp}}^{2}}, \end{array}$$

where *σ*_cp_ denotes the spread parameter for the cooperative network. In unweighted cooperation-controlled learning, information on input units is not considered, and the firing probabilities of input units are computed by

(16) $$\begin{array}{c} p\left(k\right)=\frac{1}{L}. \end{array}$$

### 2.6. Weighted Cooperation-Controlled Learning

In unweighted cooperation-controlled learning, no attention is paid to input units; however, in weighted cooperation-controlled learning, information on input units is indeed taken into account [32]. For detecting the importance of input units, we use enhanced information [33], which refers to mutual information where special attention is paid to a specific element in a network. We here consider enhanced information when attention is paid to a specific input unit.

Now we consider the case where the *t*th input unit is a target for enhancement. With this enhancement, we have the firing probability of the *j*th competitive computed by

(17) $$\begin{array}{c} p\left(j\;|\;s;t\right)=\frac{\exp\left\{-\left(1/2\right){\left(x^{s}-w_{j}\right)}^{T}\Lambda_{t}\left(x^{s}-w_{j}\right)\right\}}{\sum_{m=1}^{M}\exp\left\{-\left(1/2\right){\left(x^{s}-w_{m}\right)}^{T}\Lambda_{t}\left(x^{s}-w_{m}\right)\right\}}, \end{array}$$

where the *kl*th element of the scaling matrix (Λ_*t*_)_*kl*_ is defined by

(18) $$\begin{array}{c} {\left(\Lambda_{t}\right)}_{kl}=\frac{\delta_{kl}\delta_{kt}}{\sigma^{2}}, \end{array}$$

and we have

(19) $$\begin{array}{c} p\left(j;t\right)=\overset{S}{\underset{s=1}{\sum }}p\left(s\right)p\left(j\;|\;s;t\right). \end{array}$$

By using these probabilities, we have enhanced information for the *t*th input unit

(20) $$\begin{array}{c} {\text{EI}}_{t}=\overset{S}{\underset{s=1}{\sum }}\;\overset{M}{\underset{j=1}{\sum }}p\left(s\right)p\left(j\;|\;s;t\right)\log\frac{p\left(j\;|\;s;t\right)}{p\left(j;t\right)}. \end{array}$$

We normalize this enhanced information by

(21) $$\begin{array}{c} r\left(t\right)=\frac{{\text{EI}}_{t}}{\sum_{l=1}^{L}{\text{EI}}_{l}}. \end{array}$$

This normalized enhanced information represents the importance of input variables. As this enhanced information is increased, the *t*th input variable contributes more to the organized responses of competitive units to input patterns. In weighted cooperation-controlled learning, this enhanced information is used to estimate the firing probabilities of input units, namely,

(22) $$\begin{array}{c} p\left(k\right)=r\left(k\right). \end{array}$$

In evaluating the experimental results, we use the information content contained in input units or input information, which is defined by

(23) $$\begin{array}{c} I=\log L+\overset{L}{\underset{k=1}{\sum }}p\left(k\right)\log p\left(k\right), \end{array}$$

where *L* is the number of input units. As this input information is increased, fewer input units tend to fire. On the other hand, when the input information is small, all input units tend to fire equally.

### 2.7. Computational Methods

In determining the parameters in cooperation-controlled learning, we tried to show the effectiveness of interaction between the two types of neurons. Because the degree of interaction is determined by the interaction parameter *α*, we in particular focused on how the performance could be changed by manipulating this parameter's value. For easy comparison with the conventional SOM, we first tried to realize final networks whose performance was close to that of the conventional SOM. Then, we changed the interaction parameter *α* to examine how it would affect performance both quantitatively and visually. In addition, we tried to include as many computational techniques as possible developed for the conventional SOM to facilitate the parameter tuning and to easily compare our method to the conventional SOM.

**Figure 2:**
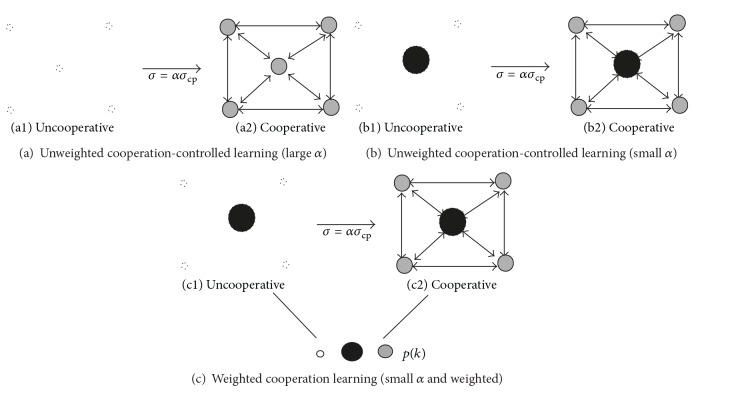
Unweighted (a) and weighted (b) cooperation-controlled learning and the parameter *α*.

Second, keeping the parameter *β*, we tried to change the other parameter *α* by using unweighted cooperation-controlled learning, as shown in Figures 2(a) and 2(b). In unweighted cooperation-controlled learning, all input units are supposed to fire equally. Then, we used the weighted cooperation-controlled learning shown in Figure 2(c), in which information on input units is included. In both methods, we carefully examined how the performance could be changed quantitatively and visually.

#### 2.7.1. Determination of the Parameter *β*

First, we tried to determine the value of the parameter *β*. To do so, we used simplified cooperative learning, because it is closest to the conventional SOM. In simplified cooperative learning, neurons are collectively treated, and only cooperation is considered. Connection weights are described by

(24) $$\begin{array}{c} w_{j}=\frac{\sum_{s=1}^{S}q\left(j\;|\;s\right)x^{s}}{\sum_{s=1}^{S}q\left(j\;|\;s\right)}. \end{array}$$

In this phase, we try to use the computational procedures developed for the conventional SOM as much as possible.

The actual parameter for the cooperation is the spread parameter *σ*_cp_ and is defined by

(25) $$\begin{array}{c} \sigma_{\text{cp}}=\frac{1}{\beta }, \end{array}$$

where *β* is larger than zero. When the parameter *β* is gradually increased, the spread parameter *σ*_cp_ gradually decreases and possibly reaches its stable points, because the increment becomes smaller. When the parameter *β* is larger, the competition becomes more like the winner-take-all; and when the parameter is small, the competition becomes soft competition.

#### 2.7.2. Cooperation Parameter *α*

In cooperation-controlled learning, we must take into account individual as well as collective neurons, namely, cooperative and uncooperative networks. In simplified cooperative learning, the parameter *β*, or the spread parameter *σ*_cp_ for cooperative networks, is determined. We thus must determine the spread parameter *σ* for the uncooperative networks. For simplicity's sake, we suppose that the spread parameter *σ* is proportional to the other parameter *σ*_cp_. Given this, we have the relation

(26) $$\begin{array}{c} \sigma =\alpha \sigma_{\text{cp}}, \end{array}$$

where *α* is called the “cooperation” parameter and is greater than zero. When the cooperation parameter *α* is sufficiently large, as shown in Figure 2(a), all competitive units respond almost equally to input patterns, meaning that the uncooperative networks have no influence on cooperative networks. On the other hand, when the cooperation parameter *α* is sufficiently small, competitive units respond very selectively to input patterns, as shown in Figure 2(b). Thus, in this case, uncooperative networks have great influence on cooperative networks. In all these methods, we suppose that input units fire equally to input patterns.

In the weighted cooperation-controlled learning presented in Figure 2(c), we have two phases. In the first phase, by using the unweighted cooperation-controlled learning, connection weights are computed along with the firing probabilities of the input units, *p*(*k*). As explained in Section 2.6, normalized enhanced information is used to approximate the firing probabilities. These probabilities are then used to compute the connection weights in the weighted cooperation-controlled learning, shown in Figure 2(c).

## 3. Results and Discussion

### 3.1. Experimental Setting

Here we present experimental results for the automobile and housing data from the machine learning database (http://archive.ics.uci.edu/ml/) to show how well our method performs. We use the SOM toolbox developed by Vesanto et al. [34] because of its simplicity in reproducing the final results presented in this paper. For SOMs, the Batch method is used, since it has shown better performance than the popular real-time method in terms of visualization, quantization, and topographic errors. Quantization errors are simply the average distance from each data vector to its BMU (best-matching unit). Topographic errors are the percentage of data vectors for which the BMU and the second-BMU are not neighboring units [19]. We should outline the evaluation measures used in this paper. To evaluate the validity of the final results, we tried to use conventional methods for easy reproduction. Three well-known conventional measures exist, namely, the topographic error [19], topographic function [20], and topographic product [21]. We chose the topographic error measure because it is easily implemented and its computational procedure is simple. For example, the topographic error produces a single value, while the topological function produces multiple values. Although the topographic product produces a single value, its computational procedure seems to be complex. More modern techniques, such as trustworthiness and continuity [23, 35], might be useful for further research, but there have been some reports on their usefulness regarding reproduction of results [19].

### 3.2. Automobile Data

#### 3.2.1. Objective and Procedures of Experiments

**Figure 3:**
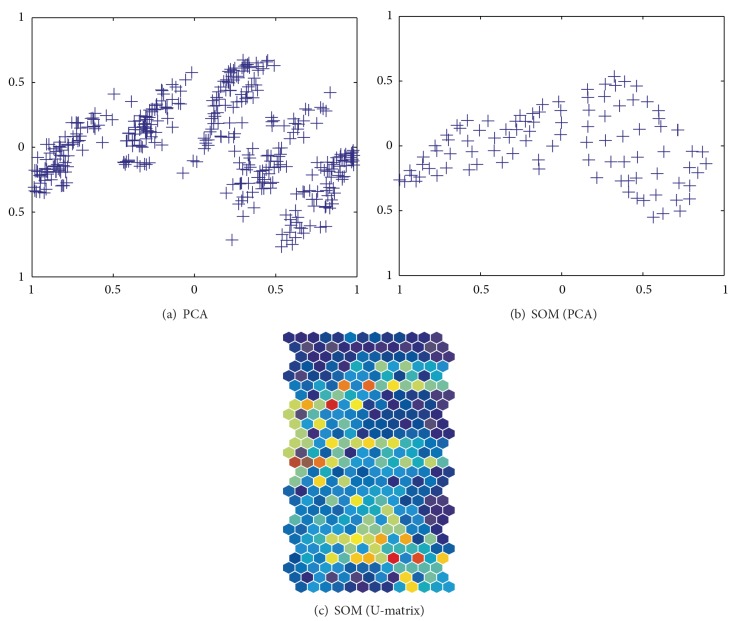
Results of PCA applied to the data (a) and connection weights by SOM (b) and the results in terms of U-matrix (c).

Here we present experimental results on the automobile data from the machine learning database to show how well our method performs. The numbers of input units and patterns were 9 and 398, respectively.  Figure 3(a) shows the results of PCA applied to the automobile data, where five classes were clearly distinguished. On the other hand, clear class boundaries could not be observed in the results of PCA applied to connection weights by the conventional SOM in Figure 3(b). In terms of the U-matrix in Figure 3(c), class boundaries in warmer colors could be seen in the middle of the map and on the upper and lower sides of the map. However, these were far from being explicit class boundaries. We thus try to show in this section how well our method could improve visualization performance by two methods, namely, unweighted and weighted cooperation-controlled learning. First, we present results obtained by the unweighted and weighted cooperation-controlled learning by changing the cooperation parameter *α*. Then, we show that the performance can be improved by using the weighted cooperation-controlled learning in terms of quantization and topographic errors.

#### 3.2.2. Unweighted Cooperation-Controlled Learning

**Figure 4:**
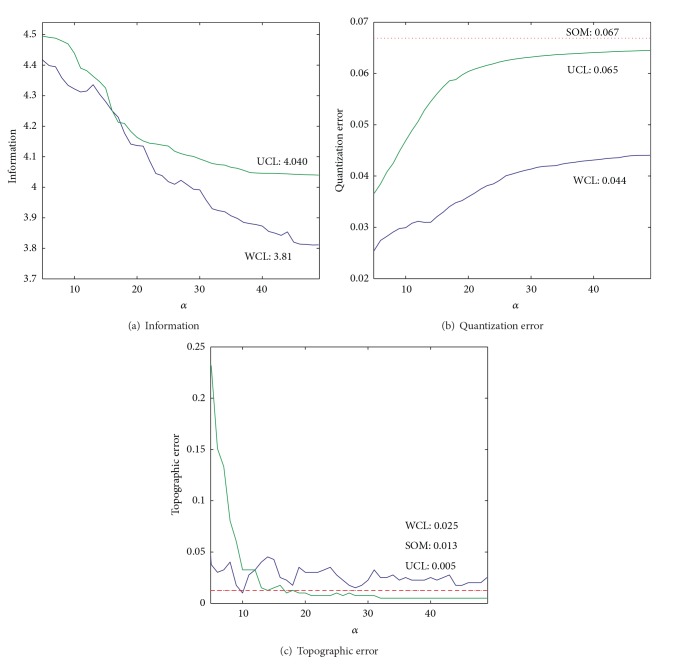
Mutual information (a), quantization (b), and topographic error (c) as a function of the cooperation parameter *α* obtained by two methods for the automobile data.

The cooperation parameter *α* was changed gradually, but the parameter *β* was fixed to 156, the value which was obtained by the simplified cooperative learning. The cooperation parameter was increased to a point where mutual information became stable.  Figure 4 shows mutual information (a), quantization errors (b), and topographic errors (c) when the cooperation parameter *α* was increased from one to 49.  Figure 4(a) shows mutual information where, by the unweighted cooperation-controlled learning, mutual information was gradually deceased and reached its stable point of 4.040.  Figure 4(b) shows quantization errors as a function of the parameter *α*. With the unweighted cooperation-controlled learning, the quantization error was increased gradually and reached a stable point of 0.065, which was slightly below the level obtained by the SOM (0.067).  Figure 4(c) shows the topographic error as a function of the parameter *α*. As the parameter was increased, the topographic error obtained by the unweighted cooperation-controlled learning decreased to 0.005, which was lower than that obtained by the conventional SOM (0.013).

**Figure 5:**
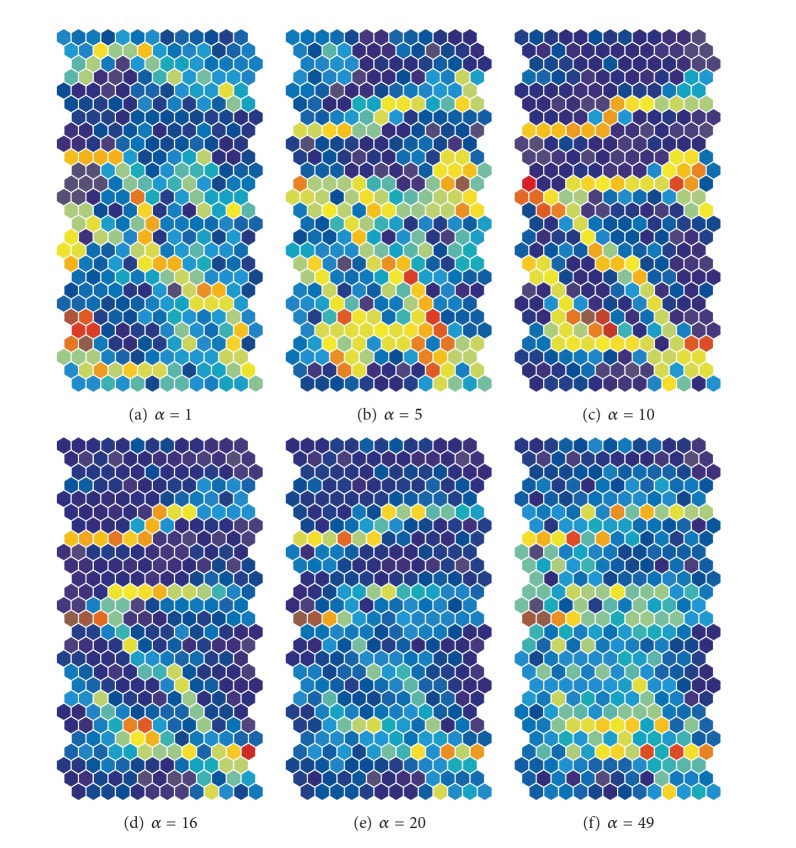
U-matrices obtained by the unweighted cooperation-controlled learning for the automobile data when the cooperation parameter was changed from 1 (a) to 49 (f).

Figure 5 shows U-matrices when the cooperation parameter *α* was increased from 1 (a) to 49 (f). When the parameter was 1, a clear diagonal class boundary could be seen, as in Figure 5(a). When the parameter *α* was increased to 5, as shown in Figure 5(b), the class boundaries classified input patterns into at least six classes. When the cooperation parameter *α* was increased to 10, class boundaries classified input patterns into five classes, as in Figure 5(c). When the cooperation parameter *α* was 16, as shown in Figure 5(d), and, as seen in Figure 9(a), when the input information was at its maximum value, clear class boundaries on the lower part of the map in Figure 5(c) became ambiguous. When the cooperation parameter *α* was increased from 20, as shown in Figure 5(e), to 49 (f), as shown in Figure 5, boundaries became very ambiguous and similar to those by the conventional SOM (shown in Figure 3(c)).

**Figure 6:**
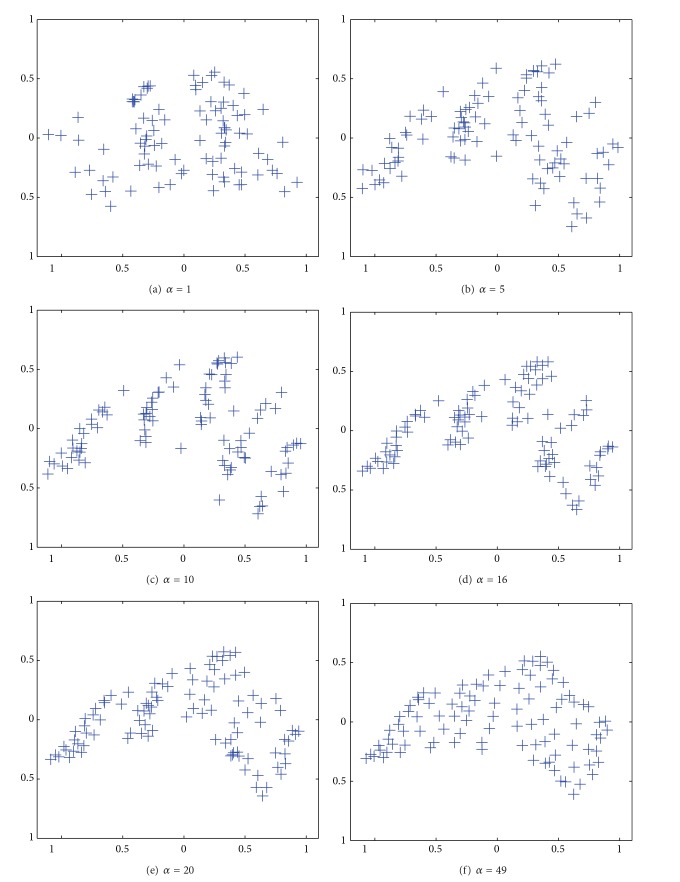
Results of PCA applied to connection weights by unweighted cooperation controlled learning when the cooperation parameter *α* was changed from 1 (a) to 49 (f).

Figure 6 shows the results of the PCA applied to connection weights by the unweighted cooperation-controlled learning. When the parameter *α* was 1, as in Figure 6(a), three classes were faintly observed. When the parameter *α* was increased to 5, as in Figure 6(b), five classes began to be separated, though class boundaries were still ambiguous. When the parameter *β* was increased to 10 in Figure 6(c) and 16 in Figure 6(d), the clearest class boundaries were observed. Then, when the parameter *α* was further increased from 20 in Figure 6(e) to 49 in Figure 6(f), these distinct class boundaries gradually disappeared.

#### 3.2.3. Weighted Cooperation-Controlled Learning

We then used the weighted cooperation-controlled learning by incorporating information on input units.  Figure 4(a) shows that, by the weighted cooperation-controlled learning, mutual information decreased and reached its stable point of 3.810, which was below the level (4.040) obtained by the unweighted cooperation-controlled learning as well as simple cooperative learning (4.021).  Figure 4(b) shows the quantization error as a function of the parameter *α*. With the weighted cooperation-controlled learning, the quantization error increased to 0.044, which was below the levels obtained by all the other methods.  Figure 4(c) shows the topographic error as a function of the parameter *α*. The topographic error obtained by the weighted cooperation-controlled learning was decreased, but the final error value was higher than that obtained by the conventional SOM.

**Figure 7:**
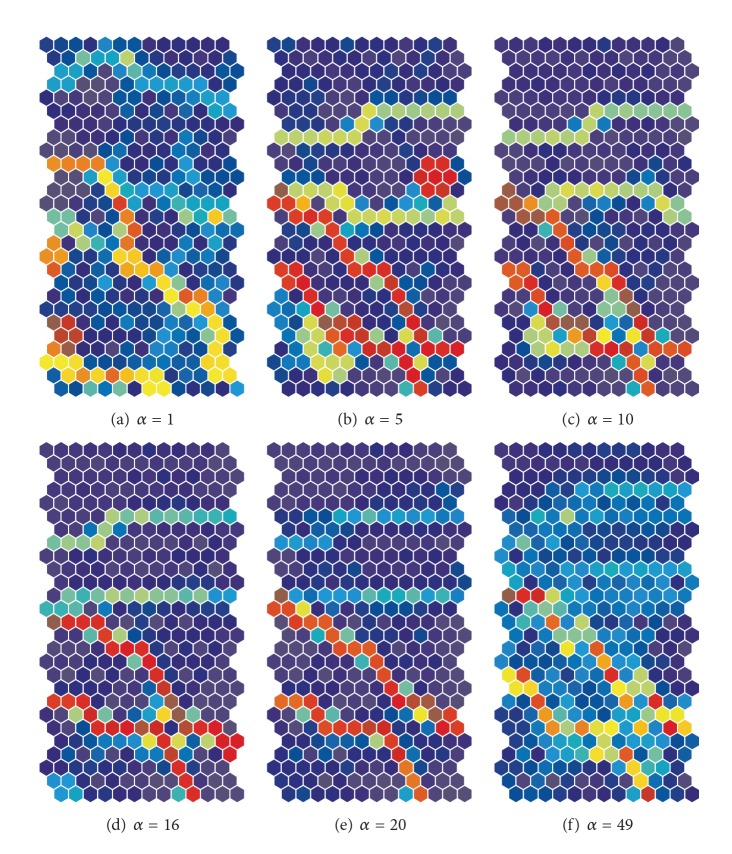
U-matrices for the automobile data obtained by the weighted cooperation-controlled learning when the cooperation parameter *α* was changed from 1 (a) to 49 (f).

Figure 7 shows the U-matrices obtained from the weighted cooperation-controlled learning when the cooperation parameter *α* was changed from 1 (a) to 49 (f). When the cooperation parameter was 1, as in Figure 7(a), a clear and diagonal boundary appeared; a boundary much clearer than the one obtained by the unweighted cooperation-learning in Figure 5(a). When the cooperation parameter *α* was increased to 5, as shown in Figure 7(b), and to 10, as shown in Figure 7(c), four clear boundaries classified input patterns into five classes, though they were slightly distorted. We can point out that the two boundaries on the lower side of the map are much clearer than the two boundaries on the upper side of the map.  Figure 7(d) shows the U-matrices when the cooperation parameter was changed to 16. The class boundaries in warmer colors became much more explicit; in addition, we could see that two boundaries on the lower side of the map were in much warmer colors. When the cooperation parameter was 20, the two class boundaries on the upper side of the map in Figure 7(e) became obscure. When the cooperation parameter was increased from 30, as shown in Figure 7(e), to 49, as shown in Figure 7(f), the two boundaries on the upper side of the map and the two diagonal positions on the lower side of the map became more obscure.

**Figure 8:**
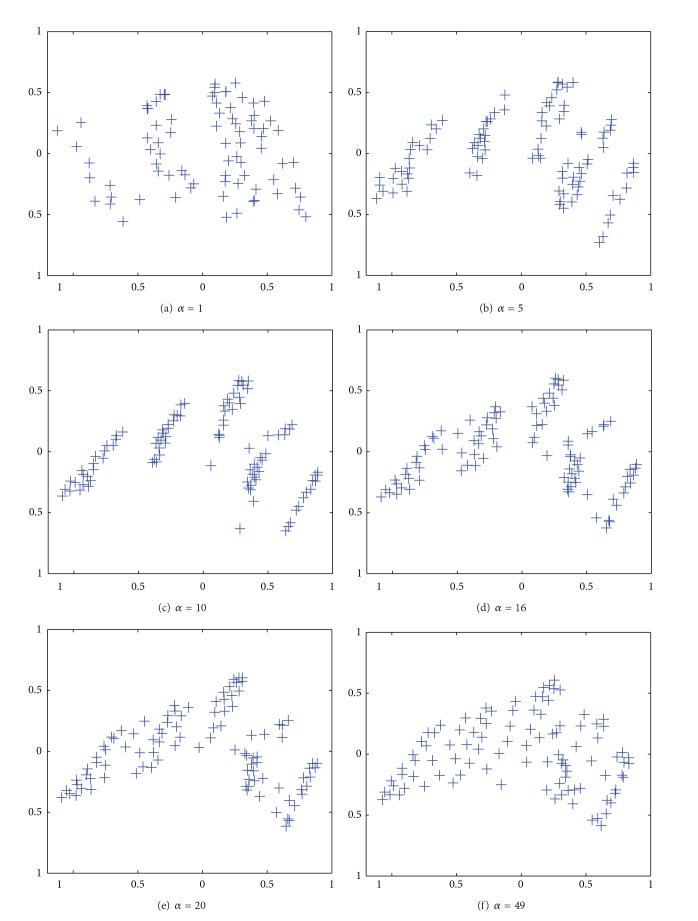
Results of PCA applied to connection weights when the parameter *α* was increased from 1 (a) to 49 (f) for the automobile data.

Figure 8(a) shows the results by the PCA when the parameter *α* was 1. Here, we were able to see three classes with some difficulty. When the parameter *α* was increased to 5 in Figure 8(b), five classes began to appear. When the parameter *α* was 10 in Figure 8(c), the clearest class boundaries divided the data into five classes. When the parameter *α* was further increased from 16 in Figure 8(d) to 49 in Figure 8(f), the class boundaries gradually disappeared.

**Figure 9:**
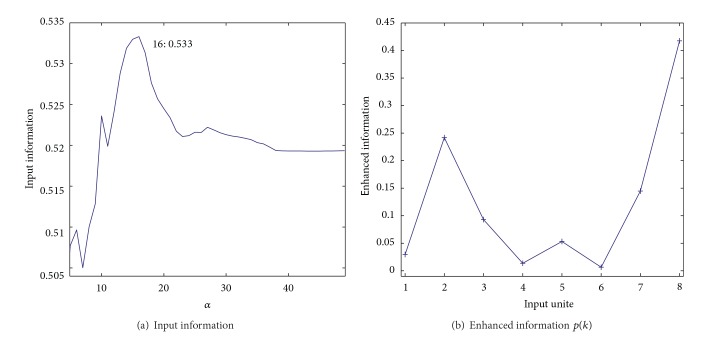
Input information in (23) (a) and enhanced information in (20) (b) for the automobile data.

**Figure 10:**
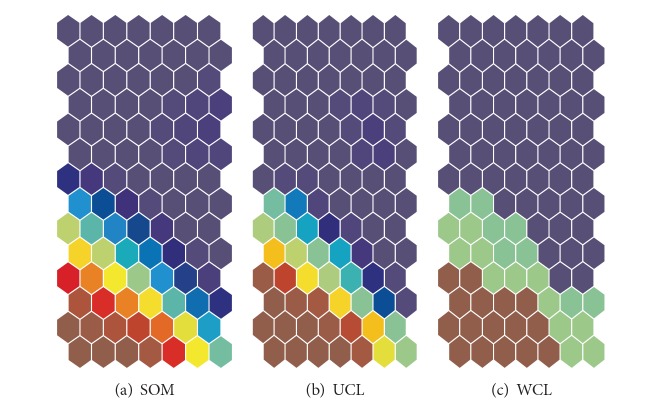
Connection weights into the eighth input unit for the automobile data by the three methods.

Figure 9(a) shows input information as a function of the parameter *α*. Though the range of input information was very small, we noticed that when the parameter was 16, the input information reached its largest value of 0.533.  Figure 9(b) shows enhanced information for eight input units (variables) when the cooperation parameter *α* was 16. As can be seen in the figure, input unit number 8 had by far the largest enhanced information, meaning that input variable number 8 played the most important role in weighted cooperation-controlled learning.  Figure 10 shows connection weights into the eighth input unit by the three methods. The connection weights became stronger on the lower left-hand side with the conventional SOM (a) and the unweighted cooperation-controlled learning (b). However, by using the weighted cooperation-controlled learning, connection weights in Figure 10(c) were clearly divided into three parts. These connection weights into the eight input unit contributed to the generation of an explicit U-matrix by the weighted cooperation-controlled learning.

#### 3.2.4. Quantitative Performance Comparison

We have shown that explicit class boundaries could be generated by changing the cooperation parameter *α*. In this section, we show that the weighted cooperative learning shows better performance in terms of quantization and topographic errors. All values were obtained when mutual information or input information reached its stable point. In addition, for statistical reliability, we chose random initialization and averaged the obtained values over ten different runs.  Table 1 shows the summary of mutual information, input information, and quantization and topographic errors obtained by the three methods. As can be seen in the table, mutual information obtained by the unweighted cooperation-controlled learning (UCL) decreased very slowly as the cooperation parameter *α* was increased. On the other hand, mutual information obtained by the weighted cooperation-controlled learning (WCL) became much smaller and reached its lowest point of 3.749 when the cooperation parameter *α* was 50. The input information obtained by the weighted cooperation-controlled learning was relatively stable at around 0.55. The quantization error obtained by the conventional SOM was 0.068. With the unweighted cooperation-controlled learning (UCL), the quantization error decreased to 0.048 when the cooperation parameter was 10. When the cooperation parameter was increased, the error gradually increased as well. With the conventional SOM, the topographic error was 0.040. With the unweighted cooperation-controlled learning, the lowest topographic error was 0.030 when the cooperation parameter was 30. On the other hand, with the weighted cooperation-controlled learning, the best error of 0.025 was obtained when the cooperation parameter was 50. Thus, in addition to generating clear class boundaries, weighted cooperation-controlled learning showed better performance in terms of quantization and topographic errors, scarifying explicit class boundaries.

### 3.3. Housing Data

#### 3.3.1. Objective and Procedures of Experiments

**Figure 11:**
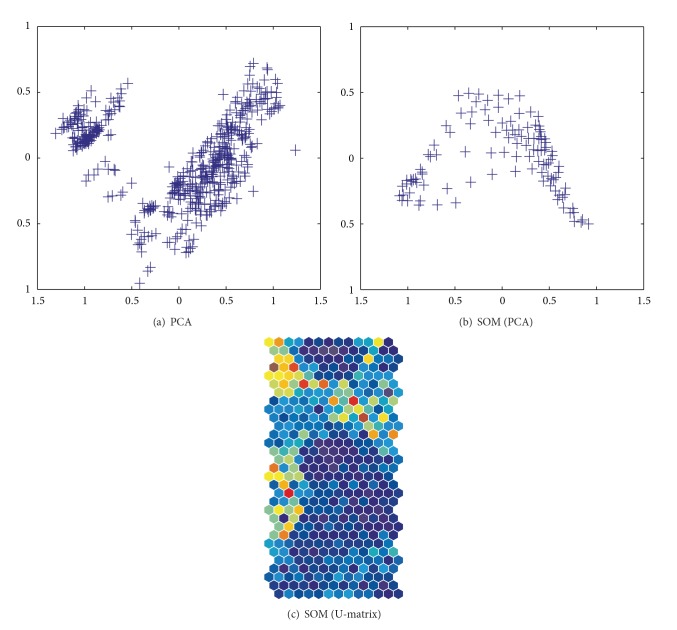
Results of PCA applied to the housing data (a), connection weights by the SOM (b), and the U-matrix by the SOM for the housing data (c).

We also applied the method to the housing data from the machine learning database. The numbers of input units and patterns were 14 and 506, respectively.  Figure 11(a) shows the results of PCA applied directly to the data itself. Two classes were distinguished, surrounded by some scattered data around them. On the other hand, when using the conventional SOM, two classes were not explicitly separated (see Figure 11(b)) in terms of the results by the PCA to connection weights. On the U-matrix in Figure 11(c), two class boundaries in warmer colors on the upper left hand side of the matrix could be seen, though they were not very clear.

We here present results obtained by the unweighted and weighted cooperation-controlled learning. In particular, we stress that clear class boundaries could be produced by the weighted cooperation-controlled learning. Finally, we also show that general performance was improved using the weighted cooperation-controlled learning.

#### 3.3.2. Unweighted Cooperation-Controlled Learning

**Figure 12:**
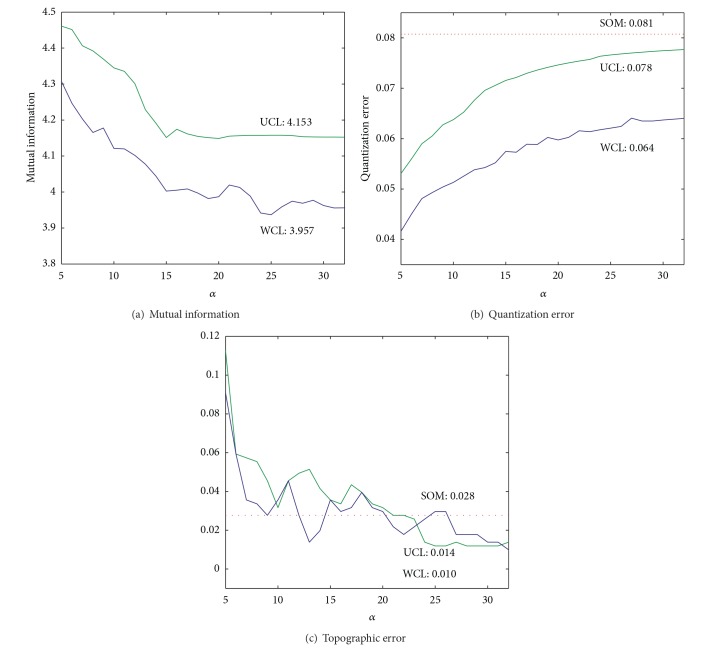
Mutual information (a), quantization (b), and topographic error (c) for input units and the cooperation parameter *α* obtained by the three methods for the housing data.

We applied the unweighted cooperation-controlled learning and weighted cooperation-controlled learning methods and increased the cooperation parameter *α* to 32. Beyond this point, learning became unstable.  Figure 12(a) shows mutual information between competitive units and input patterns obtained by the three methods. With the unweighted cooperation-controlled learning, mutual information was decreased to 4.153.  Figure 12(b) shows the quantization error as a function of the parameter *α*. With the unweighted cooperation-controlled learning, the quantization error gradually increased and reached its stable point of 0.078, which was slightly lower than the 0.081 obtained by the conventional SOM.  Figure 12(c) shows the topographic error as a function of the parameter *α*. The topographic error gradually decreased to 0.014 using unweighted cooperation-controlled learning, which was much smaller than the 0.028 obtained by the conventional SOM.

**Figure 13:**
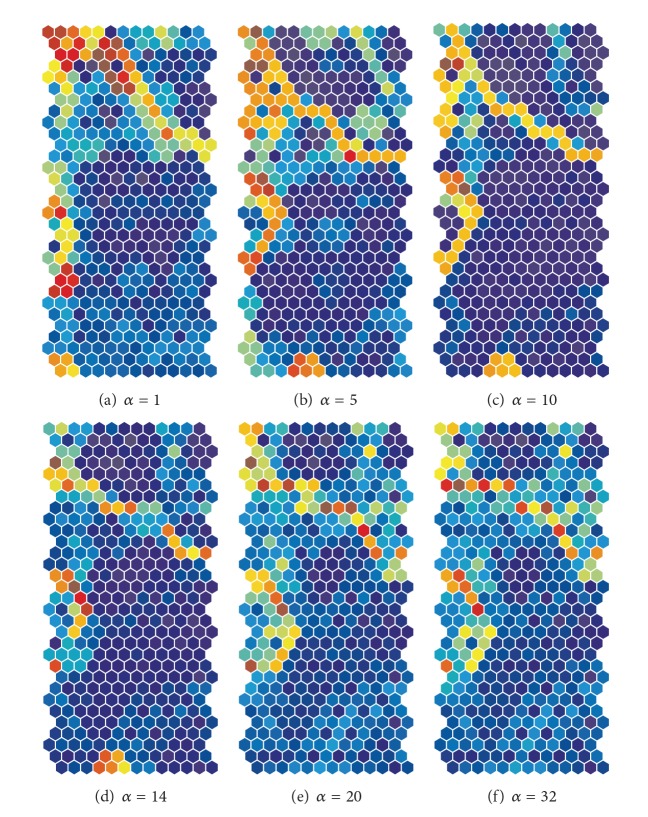
U-matrices for the housing data obtained by the unweighted cooperation-controlled learning when the cooperation parameter *α* was increased from 1 (a) to 32 (f).

Figure 13 shows the U-matrices obtained by the unweighted cooperation-controlled learning when the parameter *α* was increased from 1 (a) to 32 (f). When the cooperation parameter *α* was 1, one class boundary in warmer colors on the upper side of the U-matrix appeared; see Figure 13(a). When the cooperation parameter *α* was 5, two class boundaries on the upper side and on the left-hand side of the U-matrix appeared, as shown in Figure 13(b). When the parameter was increased from 10 to 14, as shown in Figures 13(c) and 13(d), these class boundaries became clearer. When the parameter was further increased from 20 to 32, as shown in Figures 13(e) to 13(f), the two boundaries became slightly obscure.

**Figure 14:**
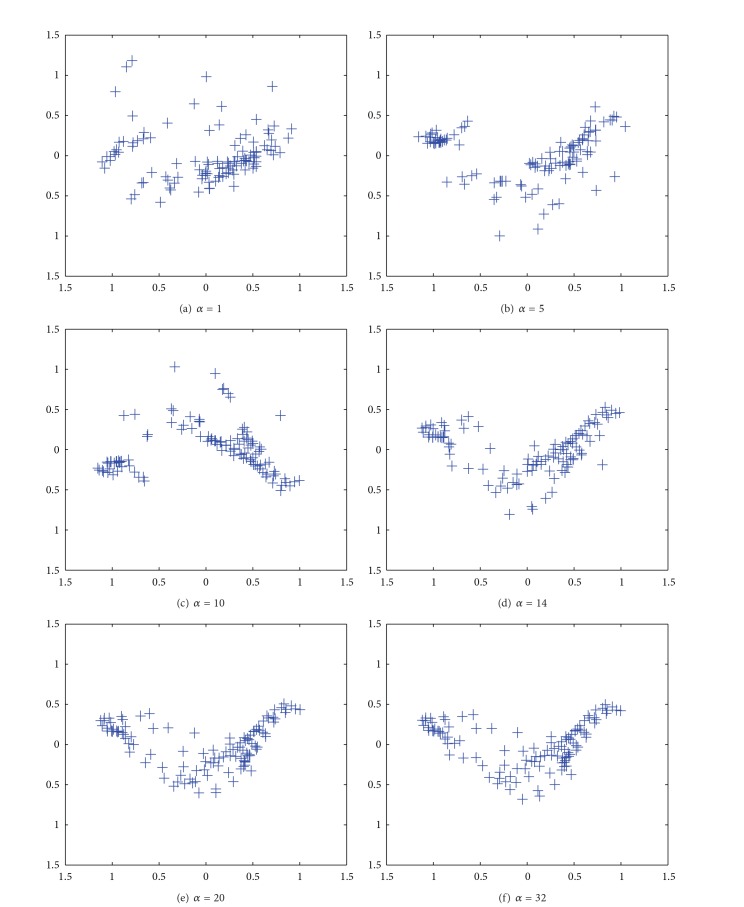
Results of PCA applied to connection weights by unweighted cooperation controlled learning when the cooperation parameter *α* was increased from one (a) to 32 (f).

Figure 14 shows the results by the PCA applied to connection weights by the unweighted cooperation-controlled learning. When the parameter *α* was 1 in Figure 14(a), connection weights were scattered widely over the map. When the parameter *β* was increased from 5 in Figure 14(b) to 10 in Figure 14(c), two classes appeared, surrounded by scatted connection weights. When the parameter *α* was further increased from 14 in Figure 14(d) to 32 in Figure 14(f), the boundary between the two classes became weaker.

#### 3.3.3. Weighted Cooperation-Controlled Learning

We then applied the weighted cooperation-controlled learning, increasing the cooperation parameter to 32.  Figure 12(a) shows mutual information between competitive units and input patterns. With the weighted cooperation-controlled learning, mutual information decreased to 3.957, which was the lowest value.  Figure 12(b) shows the quantization error as a function of the parameter *α*. With the weighted cooperation-controlled learning, the quantization error was slowly increased and reached its stable point of 0.064, which was also by far the lowest value out of all the three methods.  Figure 12(c) shows the topographic error as a function of the parameter *α*. The topographic error was gradually decreased to 0.010 by the weighted cooperation-controlled learning. This value was also the lowest one obtained by the three methods.

**Figure 15:**
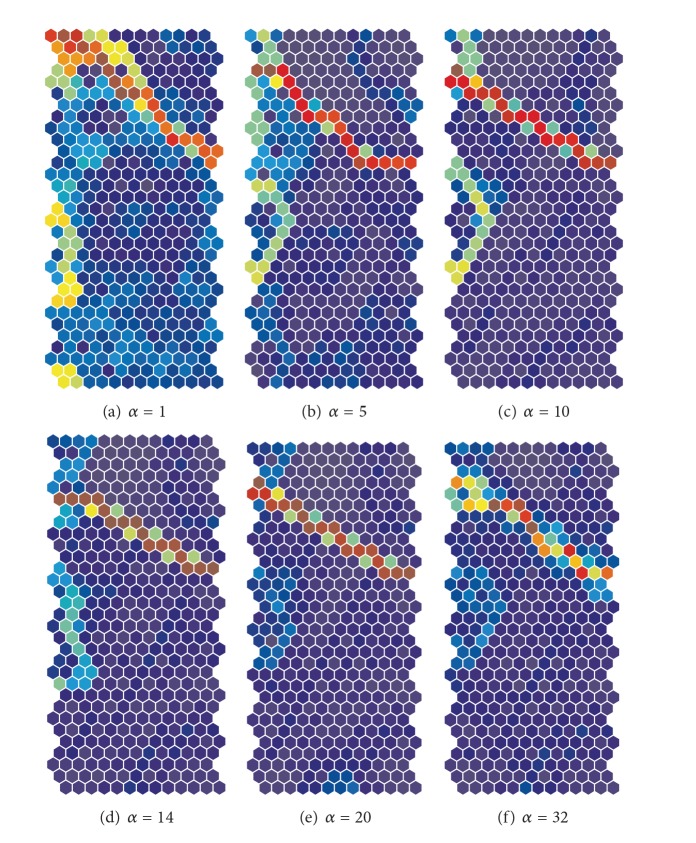
U-matrices for the housing data obtained using the weighted cooperation-controlled learning when the cooperation parameter *α* was increased from 1 (a) to 32 (f).

Figure 15 shows the U-matrices obtained by the weighted cooperation-controlled learning when the cooperation parameter was increased from 1 (a) to 32 (f). We could immediately see that all U-matrices in Figure 15 showed much more explicit class boundaries in warmer colors than those obtained by the unweighted cooperation-controlled learning, shown in Figure 13, and by SOM, shown in Figure 11. When the cooperation parameter *α* was 1, we saw one class boundary in warmer colors (see Figure 15(a)), but it was slightly distorted. When the cooperation parameter was increased to 5, the boundary on the upper side of the matrix in Figure 15(b) became more explicit. In addition, a minor boundary appeared on the left-hand side. When the parameter was increased to 10, two boundaries, shown in Figure 15(c), became more explicit. When the parameter was increased to 14, the boundary on the left-hand side, in Figure 15(d), became slightly obscure. When the parameter was increased to 20, as shown in Figure 15(e), only one class boundary in brown could be seen. Finally, when the parameter was increased to 32, the one class boundary deteriorated; see Figure 15(f).

**Figure 16:**
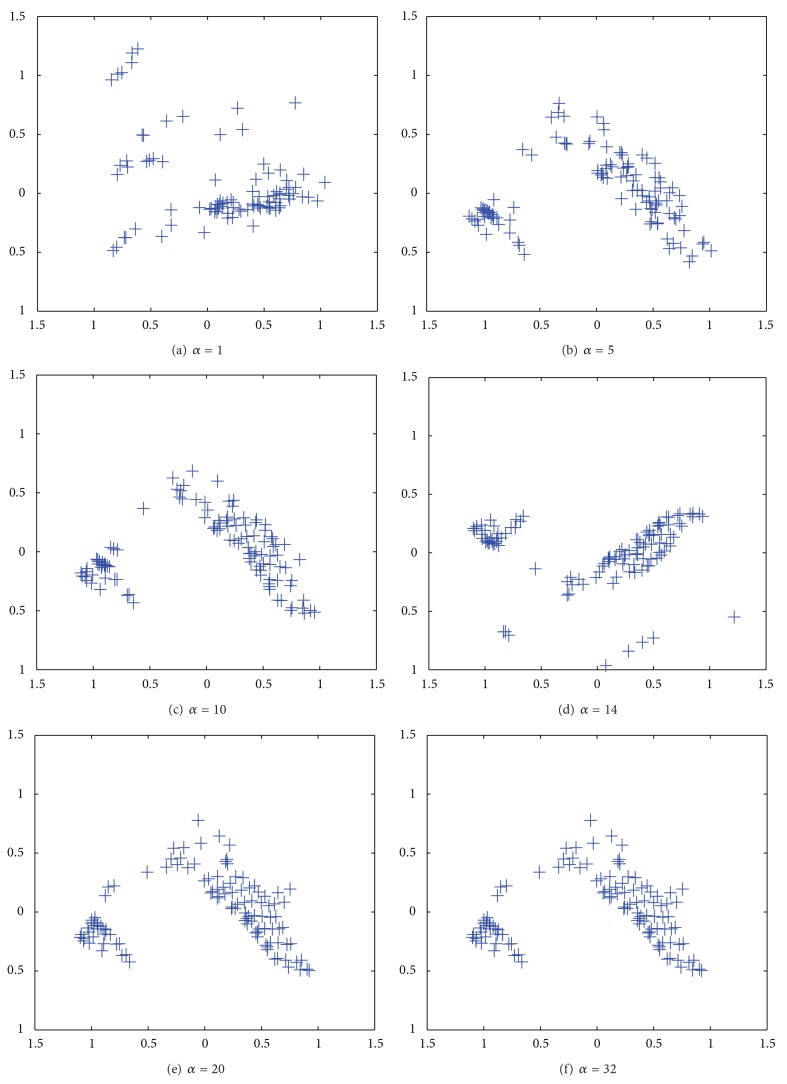
Results of PCA applied to cooperation controlled learning resolution when the parameter *α* was increased from 1 (a) to 32 (f) for the housing data.

Figure 16 shows the results of PCA applied to connection weights by the weighted cooperation-controlled learning. When the parameter *α* was 1, in Figure 16(a), connection weights were scattered over the whole map. However, when the parameter *α* was increased from 5 in Figure 16(b), to 10 in Figure 16(c), two classes were clearly formed. In particular, when the parameter *α* was 10, in Figure 16(c), the best results were obtained. When the parameter *α* was further increased from 14 in Figure 16(d) to 32 in Figure 16(f), the two classes slightly began to deteriorate.

**Figure 17:**
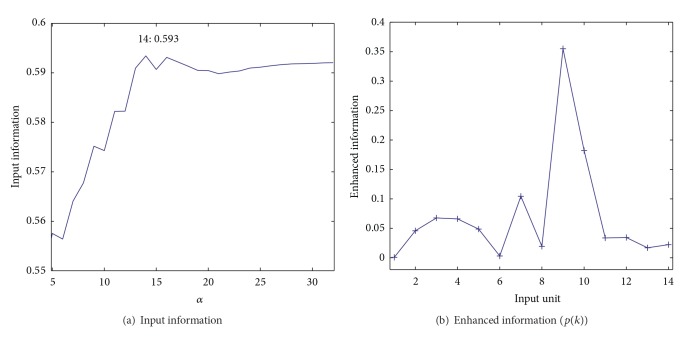
Input information in (23) (a) and enhanced information in (20) (b) for the housing data.

**Figure 18:**
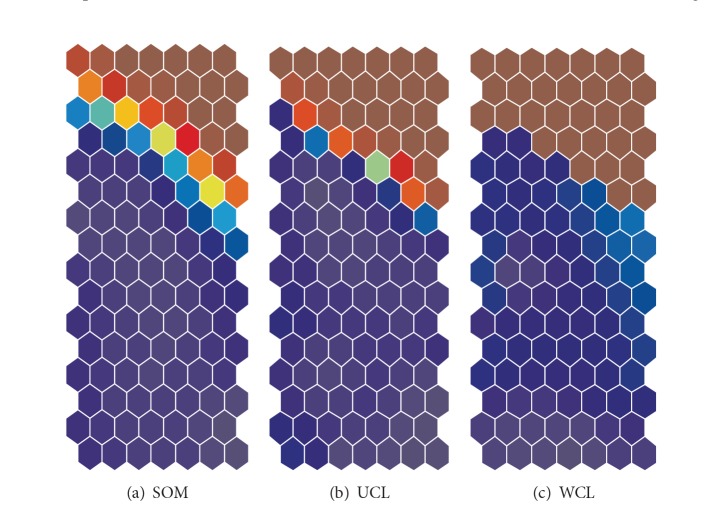
Connection weights or component planes along input unit number 9 for the housing data when the cooperation parameter is 14 with maximum input information.

Figure 17(a) shows input information as a function of the parameter *α*. When the parameter was 14, the input information reached its highest point of 0.593.  Figure 17(b) shows enhanced information for fourteen input units (variables). As can be seen in the figure, the enhanced information for input unit number 9 had the highest value, meaning that the ninth input unit played the most important role.  Figure 18 shows connection weights into the ninth input unit obtained by the three methods. When the conventional SOM was used, two parts in Figure 18(a) were separated, with some distortion on the boundary. In the cooperation-controlled learning, shown in Figure 18(b), the boundary became more explicit. Finally, with the weighted cooperation-controlled learning, two parts were the most clearly separated; see Figure 18(c).

#### 3.3.4. Improved Performance

We also showed improved performance in terms of the precision of the quantization error and continuity of the topographic error. All values were obtained when mutual information or input information reached its steady state.  Table 2 shows the summary of mutual information, input information, and quantization and topographic errors obtained by the three methods. As can be seen in the table, mutual information obtained by the cooperation-controlled learning decreased very slowly as the cooperation parameter *α* was increased. On the other hand, mutual information obtained by the weighted cooperation-controlled learning became much smaller and reached its lowest point of 3.909 when the cooperation parameter *α* was 30. The input information obtained by the weighted cooperation-controlled learning was 0.508 when the cooperation parameter was 5; at this point, the input information gradually increased and became stable. The quantization error obtained by the conventional SOM was 0.081. With the unweighted cooperation-controlled learning, the error further decreased to 0.052 when the cooperation parameter was 5. Then, the error gradually increased to 0.078 when the cooperation parameter was 30. With the weighted cooperation-controlled learning, the quantization error further decreased to 0.042, and the error increased up to 0.063 when the cooperation parameter was 30. The topographic error obtained by the conventional SOM was 0.027. With the unweighted cooperation-controlled learning, the topographic error also slightly decreased to 0.025 when the cooperation parameter was 30. With the weighted cooperation-controlled learning, the topographic error further decreased and reached its smallest point of 0.021 when the cooperation parameter was 30. Thus, we can say that in terms of precision and continuity, the best performance was obtained by the weighted cooperation-controlled learning.

### 3.4. Discussion

#### 3.4.1. Validity of Methods and Experimental Results

Here, we summarize and discuss the final results in terms of errors and general performance. In particular, we discuss why improved performance in terms of quantization and topographic errors can be obtained from different points of view.

First, experimental results can be summarized in terms of quantization and topographic errors. The quantization error is increased when the cooperation parameter *α* is increased, as shown in Figures 4(b) and 12(b). In other words, as the cooperative networks become dominant, the quantization errors become larger. Inversely, as uncooperative networks become dominant, quantization errors are decreased. Second, the topographic error becomes smaller as the cooperative parameter is increased, as shown in Figures 4(c) and 12(c). This is natural because the cooperation parameter controls the degree of cooperation. When the cooperation parameter *α* is increased, maps should be more topologically organized. These results show that the cooperation parameter *α* is effective at controlling the two types of errors.

Second, we have shown that class boundaries are experimentally made clearer with the cooperation-controlled learning, particularly with the use of weighted cooperation-controlled learning. The reason why such clearer class boundaries are generated can be conjectured as follows. In the conventional SOM, cooperation plays one of the most important roles, and neighboring neurons are trained to behave or fire in a similar way. Thus, even if there exists a class boundary, neighboring neurons still fire in the same way, which makes it difficult to see class boundaries on the map. However, it is possible to take into account the effect of uncooperative networks, which makes the class boundaries more easily detected.

Third, cooperation-controlled learning can be used to improve the general performance of networks. We have shown in the sections on quantitative performance comparison that improved performance can be obtained using weighted cooperation-controlled learning. Our inference regarding this good performance is based upon the mutual information obtained by learning. We observed in Figures 4 and 12 that mutual information obtained by the weighted cooperation-controlled learning is always below the values of mutual information obtained by the unweighted cooperation-controlled learning. Additionally, mutual information obtained by the weighted cooperation-controlled learning is smaller than that obtained by the conventional SOM when the cooperation parameter *α* is larger. Because the amount of mutual information is directly related to the information on input patterns, smaller mutual information obtained by the weighted cooperation-controlled learning shows that not much detailed information on input patterns is acquired by the weighted cooperation-controlled learning when the cooperation parameter *α* is sufficiently large, though explicit class boundaries tend to disappear.

#### 3.4.2. Limitation of the Method

Though we have shown how well our method performs, several problems can be pointed out. We summarize those problems here with two points, namely, parameter tuning and the lack of a comprehensibility measure. First, there are three parameters in the method, namely, the neighborhood parameter *σ*_nh_, the competition parameter *β*, and the cooperation parameter *α*. All three parameters should be tuned at the same time for the sake of finely controlling learning; however, it is extremely difficult to control all three parameters. In this paper, to simplify the parameter tuning, we used two steps of learning. The first step is concerned only with the cooperation where the parameter *σ*_nh_ is decreased to 1 gradually, according to the computational procedure of the conventional SOM. On the other hand, the competition parameter *β* is gradually increased up to a point where learning becomes unstable. As the competition parameter *β* becomes larger, the procedure becomes similar to that of the conventional SOM. Then, the competition parameter *β* is fixed, and the cooperation parameter *α* is increased. Thus, we need to compute connection weights for the two steps with different values of the parameters. To accelerate learning, we need to unify these two steps into one.

Second, when the cooperation parameter *α* was small, very clear class boundaries were observed in the automobile and housing data, shown in Figures 7 and 15. These clear class boundaries tended to disappear as the cooperation parameter *α* was increased. Thus, we need to know which representations are best or better. In terms of quantization errors, the representations are better when the cooperation parameter *α* is smaller, as shown in Figures 4 and 12. In terms of the topographic error, it is better when the cooperation parameter is rather larger, as shown in Tables 2 and 1. However, when the cooperation parameter is larger, class boundaries become obscure, as shown in Figures 7 and 15. Thus, we need a measure of comprehensibility to determine the best possible representation among many.

#### 3.4.3. Possibility of the Method

The possibility can be described in three ways, namely, in terms of flexibility, variety, and extension to multiple networks. The possibility of our method lies in its flexibility. As shown by the experimental results, our method is very flexible. By merely changing the cooperation parameter *α*, maps with different amounts of mutual information can be generated, from maps with explicit class boundaries to maps with less explicit ones. More minor class boundaries can be generated when mutual information is at its largest. As the cooperation parameter *α* grows smaller, the quantization error is decreased and precision is increased. On the other hand, as the cooperation parameter is increased, the topographic error is generally decreased, though explicit class boundaries tend to disappear. The magnification control method [36, 37] has produced similar types of results. The magnification parameter in the method is changed to take into account a variety of quantization properties. In addition, the minor class boundaries can be detected by the negative magnificent parameter. As was already mentioned, our method can be used to control mutual information in networks. As the mutual information is increased, more detailed information on input patterns can be accumulated in the network. This detailed information is related to the detection of class boundaries.

Secondly, a variety of targets can be implemented in our model. In our model, uncooperative networks try to imitate cooperative networks as much as possible. However, we can replace cooperative networks by any kind of network. For example, it can be replaced by a network that aims to minimize errors between targets and outputs from the networks. Thus, it is easy to realize supervised learning by using our method. If we can combine cooperation and supervised learning, it is possible to produce internal representations that are easily interpretable.

Finally, we can extend the two types of networks discussed in this paper to multiple types of networks. As mentioned, our method fundamentally tries to deal with many different types of networks by examining a network from different points of views. One of the easiest ways to produce many different networks is to change the parameters inside the neural networks. Even if different networks with different parameter values are present, they are still derived from one network; thus, we should examine the interactions between different networks. The extension to multiple points of view can be used to enhance final representations more flexibly by controlling the effect of different networks, though the computational complexity becomes large.

## 4. Conclusion

In the present paper, we have proposed a new type of information-theoretic method called “cooperation-controlled learning” in which two types of networks are supposed, namely, collective and uncollective networks. In the cooperative network, neurons are treated collectively, while in the uncooperative network neurons are treated individually. Difference between the cooperative and uncooperative networks was represented by the Kullback-Libler divergence between the two firing probabilities of each network type. All the equations, including the reestimation equation for connection weights, were derived from a minimization of the KL-divergence. In addition, the cooperation parameter *α* was introduced to control the degree of influence of the two types of networks. When the parameter *α* was increased, the effect of cooperative networks gradually began to play an important role. We applied the methods to two sets of data, namely, the automobile and housing classification data from the machine learning database. In both data sets, clearer class boundaries gradually appeared when the parameter *α* was increased. In addition, the quantization and topographic errors did not necessarily increase, despite the clear class structure obtained by our method.

Though the effectiveness of our method was confirmed only by experiments, we should in the future more explicitly and theoretically explain the reason for the improved performance. In particular, the optimal state for the most explicit representation should be theoretically determined. In addition, two types of networks should be extended to multiple networks whose interaction may produce very special effects to neural networks.

## Figures and Tables

**Table 1:** Information, input information, quantization, and topographic errors obtained by the three methods with random initialization for the automobile data. The parameter β was set to 100 to stabilize learning. In the table, “information” and “input inf” represent mutual information and input information, respectively.

Methods	Measures	α	Information	Input inf.	QE	TE
SOM	Average				0.068	0.040
	Std dev.				0.001	0.018

UCL	Average	10	4.354	0.000	0.048	0.125
	Std dev.		0.024	0.000	0.001	0.069
	Average	20	4.151	0.000	0.061	0.031
	Std dev.		0.036	0.000	0.001	0.022
	Average	30	4.099	0.000	0.064	0.030
	Std dev.		0.043	0.000	0.001	0.018
	Average	40	4.065	0.000	0.065	0.035
	Std dev.		0.044	0.000	0.001	0.014
	Average	50	4.044	0.000	0.066	0.035
	Std dev.		0.047	0.000	0.001	0.018

WCL	Average	10	4.241	0.554	** 0.030 **	0.088
	Std dev.		0.024	0.038	0.001	0.042
	Average	20	4.066	0.547	0.036	0.042
	Std dev.		0.035	0.021	0.001	0.019
	Average	30	3.861	0.560	0.041	0.028
	Std dev.		0.064	0.030	0.002	0.012
	Average	40	3.767	0.556	0.043	0.028
	Std dev.		0.046	0.03	0.002	0.014
	Average	50	3.749	0.556	0.043	** 0.025 **
	Std dev.		0.059	0.031	0.002	0.012

**Table 2:** Information, input information, and quantization and topographic errors obtained by the three methods for the housing data. The parameter β was set to 100. In the table, “information” and “input inf” represent mutual information and input information, respectively.

Methods	Measures	α	Information	Input inf.	QE	TE
SOM	Average				0.081	0.027
	Std dev.				0.001	0.012

UCL	Average	5	4.428	0.000	0.052	0.234
	Std dev.		0.038	0.000	0.001	0.077
	Average	10	4.324	0.000	0.064	0.045
	Std dev.		0.028	0.000	0.001	0.017
	Average	15	4.207	0.000	0.072	0.033
	Std dev.		0.034	0.000	0.001	0.014
	Average	20	4.12	0.000	0.076	0.030
	Std dev.		0.037	0.000	0.001	0.014
	Average	25	4.093	0.000	0.077	0.029
	Std dev.		0.027	0.000	0.001	0.011
	Average	30	4.08	0.000	0.078	0.025
	Std dev.		0.046	0.000	0.001	0.009

WCL	Average	5	4.323	0.508	** 0.042 **	0.142
	Std dev.		0.016	0.057	0.000	0.046
	Average	10	4.100	0.565	0.051	0.029
	Std dev.		0.028	0.020	0.001	0.013
	Average	15	4.023	0.583	0.056	0.031
	Std dev.		0.028	0.030	0.001	0.016
	Average	20	3.981	0.586	0.059	0.023
	Std dev.		0.023	0.032	0.001	0.008
	Average	25	3.947	0.590	0.061	** 0.021 **
	Std dev.		0.027	0.033	0.002	0.006
	Average	30	3.909	0.588	0.063	0.025
	Std dev.		0.027	0.035	0.002	0.008
